# The Impact of Closed Incision Negative Pressure Therapy on Postoperative Breast Reconstruction Outcomes

**DOI:** 10.1097/GOX.0000000000001880

**Published:** 2018-08-07

**Authors:** Allen Gabriel, Steven Sigalove, Noemi Sigalove, Toni Storm-Dickerson, Jami Rice, Patrick Maxwell, Leah Griffin

**Affiliations:** From the * PeaceHealth Medical Group, Vancouver, Wash.; † DuPage Medical Group, Glen Ellyn, Ill.; ‡ Department of Plastic Surgery and Breast Surgery, DuPage Medical Group/DMG Aesthetics, Central DuPage Hospital/Northwestern Medicine, Winfield, Ill.; § Compass Oncology, Vancouver, Wash.; ¶ Loma Linda University Medical Center, Loma Linda, Calif.; ‖ Acelity, San Antonio, Tex.

## Abstract

**Background:**

Studies report that incision management with closed incision negative pressure therapy (ciNPT) may provide clinical benefits, including protecting surgical incisions, for postsurgical closed incisions (eg, orthopedic, sternotomy, and colorectal). This retrospective analysis compared postoperative outcomes in patients who received ciNPT versus standard of care (SOC) for incision management after breast reconstruction postmastectomy.

**Methods:**

Patient demographics, chemotherapy exposure, surgical technique, ciNPT use, number of drains, time to drain removal, and 90-day postoperative complication rates were analyzed from records of 356 patients (ciNPT = 177, SOC = 179) with 665 closed breast incisions (ciNPT = 331, SOC = 334).

**Results:**

Overall complication rate was 8.5% (28/331) in ciNPT group compared with 15.9% (53/334) in SOC group (*P* = 0.0092). Compared with the SOC group, the ciNPT group had significantly lower infection rates [7/331 (2.1%) versus 15/334 (4.5%), respectively; *P* = 0.0225], dehiscence rates [8/331 (2.4%) versus 18/334 (5.4%), respectively; *P* = 0.0178], necrosis rates [17/331 (5.1%) versus 31/334 (9.3%), respectively; *P* = 0.0070], and seroma rates [6/331 (1.8%) versus 19/334 (5.7%), respectively; *P* = 0.0106]. The ciNPT group required significantly fewer returns to operating room compared with the SOC group [8/331 (2.4%) versus 18/334 (5.4%), respectively; *P* = 0.0496]. Time to complete drain removal per breast for ciNPT versus SOC groups was 9.9 versus 13.1 days (*P* < 0.0001), respectively.

**Conclusions:**

Patients who received ciNPT over closed incisions following postmastectomy breast reconstruction experienced a shorter time to drain removal and significantly lower rates of infection, dehiscence, necrosis, and seromas, compared with the SOC group. Randomized controlled studies are needed to corroborate the findings in our study.

## INTRODUCTION

The number of women seeking breast reconstruction following mastectomy has steadily risen since 2000 with a 3% increase between 2015 and 2016. In fact, more than 109,000 breast reconstructions were performed in the United States during 2016.^1^ Although it is currently estimated that just less than 20% of U.S. women who require a mastectomy choose to undergo immediate reconstruction, this rate is also rising.^1^ Implant-based breast reconstructions continue to be the most popular option, accounting for nearly 80% of procedures.^2,3^ Reasons for increased implant-based breast reconstruction procedures include no donor-site morbidity, shorter procedure times, a younger patient population, a lack of autologous donor tissue, and an increase in the number of bilateral mastectomies.^2–4^

Complication risks after breast reconstruction differ greatly depending on the approach, and the reported overall breast-prosthetic infection rates range widely from 1% to 35%.^5–9^ Although convenient, implant-based breast reconstruction procedures have higher rates of postoperative complications, whereby postoperative infection remains the most common and likely the most feared complication from breast reconstruction surgery.^10^ For example, in a database analysis of 3,007 women who received immediate implant-based reconstruction postmastectomy, infection was diagnosed in 20.5% of patients.^8^ Infection of the implant can lead to prolonged antibiotic treatment, undesired additional surgical procedures, and unsatisfactory results.^2^ Lastly, postoperative wound complications following immediate breast reconstruction have also been associated with a significantly greater risk of developing systemic recurrence of cancer.^11^ Prolonged drain use (> 3 weeks) has also been correlated with a higher rate of postoperative infection,^12,13^ and drains can be uncomfortable, inconvenient, and painful for patients. However, closed-suction drains can decrease seroma formation and possibly reduce the risk of postoperative infection.^14,15^

Evidence-based approaches aimed at minimizing the risk of postoperative wound complications in patients with breast cancer undergoing immediate reconstruction are essential in maximizing outcomes. The use of closed incision negative pressure therapy (ciNPT) has increased in recent years to help manage incisions in various surgical specialties including orthopedic,^16^ sternotomy,^17^ groin,^18^ vascular,^19^ colorectal,^20^ and abdominal surgery,^21–24^ by helping to hold the incision edges together, protecting surgical incisions from external contamination, and removing fluids and infectious materials.^25^ The aim of this study was to compare postoperative outcomes and time to drain removal among patients using ciNPT versus standard of care (SOC; steristrips) after breast reconstruction following mastectomy.

## METHODS

### Patients

Deidentified patient data were used in accordance with the principles outlined in the Declaration of Helsinki. This was a single site, retrospective review of records for adult female patients who underwent breast reconstruction postmastectomy between July 1, 2009 and October 31, 2017. Patient records were extracted for analysis if they were complete and included a 90-day follow-up period within the date range. Records of patients who received radiation at any time during breast cancer treatment were excluded from analysis. Records were then divided into 2 groups for comparative analysis: patients who received SOC versus patients who received ciNPT for closed incision management post breast reconstruction.

The decision to use SOC versus ciNPT was largely dependent on availability of ciNPT devices. Most of the patients in the SOC group were treated during the beginning years of the study timeframe (July 2009 to July 2014), before commercial availability of ciNPT devices. The vast majority of ciNPT-treated patients received care during the latter period after ciNPT was commercially available (March 2016 to October 2017). During the middle of the study period (July 2014 to February 2016), there was a period of overlap while ciNPT devices and dressings were being evaluated by the hospital, and during which time both SOC and ciNPT were used. During this time, patients received ciNPT if they had at least 1 risk factor (diabetes mellitus, high body mass index, nicotine use, soiling, radiation, or immunosuppression) associated with postoperative complications and/or if their reconstruction involved large undermined areas, use of biologics/synthetics, tight closure, a compromised flap, or a repeated incision through the same scar. Patients with no risk factors received SOC.

### Surgical Procedure

All patients underwent skin-sparing, nipple-sparing, or skin-reducing mastectomy with immediate or delayed expander-implant reconstruction involving acellular dermal matrix support. Reconstruction technique consisted of prepectoral tissue expander placement or partial submuscular/partial acellular dermal matrix (dual-plane) expander placement.^26^ Types of incisions varied between vertical, inframammary fold and transverse, but incision type and location were not tracked as variables in this retrospective review. All incisions were closed with our standard technique using absorbable Monocryl 3-0 sutures (Ethicon, Cornelia, Ga.) for the deep dermis and Monocryl 4-0 sutures for subcuticular skin closure. Drains were placed in the subcutaneous pocket. In immediately constructed breasts, two 15-French round Jackson-Pratt drains were placed laterally without crossing the breast meridian. In delayed reconstruction or revision surgery, only 1 drain was placed in the breast. For patients who received ciNPT, drains were routed under the skin beyond the dressing.

Steristrips were placed over the incision in the SOC group. For the ciNPT group, a 33-cm long foam dressing with adhesive backing (PREVENA CUSTOMIZABLE Dressing, KCI, an Acelity company, San Antonio, Tex.) was applied immediately over the closed incision in the OR, overlapping the incision line by approximately 3 cm on either side. Negative pressure therapy (PREVENA Incision Management System, KCI, an Acelity company, San Antonio, Tex.) was applied at −125 mm Hg.

All patients were discharged home after 1 midnight stay and instructed to return for follow-up on postoperative days 3 and 7. In cases of 2 drains per breast, 1 drain per breast was removed on postoperative day 3; the second drain was removed when output was less than 30 cc per 24 hours. In cases of 1 drain per breast, the drain was removed when drainage was less than 30 cc per 24 hours. ciNPT was carefully removed and discontinued on postoperative day 7, and xeroform gauze or a skin adhesive closure (STERI-STRIP Skin Closure, 3M, St. Paul, Minn.) was applied over each incision. Patients were followed up for 90 days after surgery.

### Statistical Analysis

Patient characteristics, comorbidities, chemotherapy, and surgical technique (dual plane versus prepectoral) were recorded into an Excel (Microsoft, Redmond, Wash.) database. Number of drains placed, time to drain removal, and complications within 90 days postsurgery were also recorded. Time to drain removal was rounded to the nearest day. Patients were noted to have a complication if at least one of the following occurred: surgical-site infection, dehiscence, necrosis, seroma, hematoma, tissue expander exposure, tissue expander replacement, or return to the operating room. Infection was defined by a clinical need for antibiotics as determined by the lead investigator. Analyses were performed using SAS 9.4 (SAS Institute Inc., Cary, N.C.). Continuous variables were presented as mean ± SD, whereas qualitative data were presented as number and percentage. For quantitative data, median, minima, and maxima were calculated. Categorical variables were presented as the number of patients or number of breasts and its corresponding proportion with respect to the group under study.

Propensity score stratification was used to balance the covariates in the 2 groups and eliminate biased estimates of the effect of ciNPT. The estimated propensity score was the probability of receiving either SOC or ciNPT, given 13 covariates. Patients were divided into 5 strata based on their estimated propensity score. Patient and breast characteristics were compared before propensity score stratification with *t* tests for continuous variables and Chi-square or Fisher’s exact tests for categorical variables. The same patient and breast characteristics were compared after adjusting for their propensity quintile. This was done using general linear models for continuous variables and generalized linear models for categorical variables. The models included main effects for propensity score quintile and ciNPT use. The interaction of quintile and ciNPT use was also examined. Imbalances between the SOC and ciNPT groups were reviewed after adjustment for propensity score quintile and compared with imbalances before adjustment. Outcome analyses were performed for overall complication rates, individual complication types, and time to final drain removal. The models for these analyses also included main effects for propensity score quintile and ciNPT use. The outcome analyses were then repeated with surgery type added as a covariate in addition to ciNPT use and propensity score quintile.

## RESULTS

### Patient Demographics

Records of 356 female patients (ciNPT = 177, SOC = 179) accounting for 665 reconstructed breasts (ciNPT = 331, SOC = 334) were analyzed. Demographics and types of surgery performed are listed in Table 1. The initial raw data showed significant imbalances between the 2 cohorts in age, diabetes, hypertension, expander placement timing, and reconstruction surgery type, and a marginal difference in chemotherapy. Patients in the ciNPT group were older and had higher incidences of diabetes, hypertension, and chemotherapy treatment. Delayed (versus immediate) and dual plane (versus prepectoral) expander-implant reconstruction occurred more often in the SOC group. Skin-reducing mastectomies were performed more in the ciNPT group, and skin-sparing mastectomies were performed more in the SOC group. After adjustment for propensity score quintile, imbalance remained in only 1 baseline variable: reconstruction surgery type. Far more patients in the ciNPT group received prepectoral versus dual plane surgery; this reflects our shift in practice toward prepectoral reconstruction, which occurred in the later years of the study timeframe when most ciNPT systems were used.

**Table 1.**
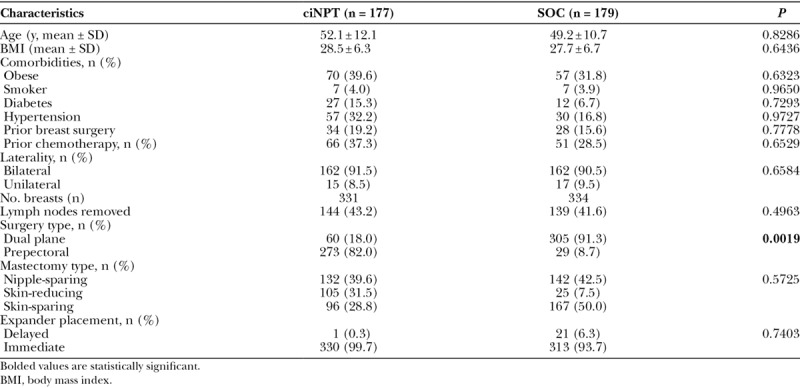
Demographics and Baseline Characteristics

### Postoperative Complication Rates

The ciNPT group had significantly lower rates of overall complications (*P* = 0.0092), including a significantly lower rate of surgical-site infection (*P* = 0.0225), dehiscence (*P* = 0.0178), necrosis (*P* = 0.0070), seroma formation (*P* = 0.0106), and returns to the operating room (*P* = 0.0496) compared with the SOC group (Table 2). There were no significant differences in the rates of postoperative hematomas, tissue expander exposure, or expander removal between the groups (Table 2). Subsequent analyses to include surgery type as a covariate in addition to propensity score quintile continued to demonstrate similar results.

**Table 2.**
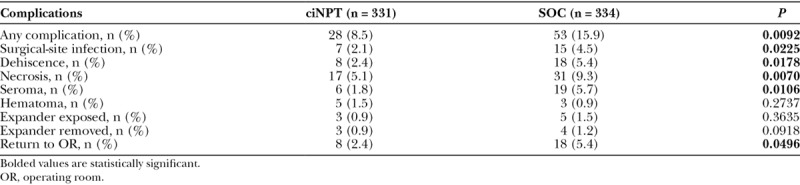
Postoperative Complication Rates of ciNPT Versus SOC Groups

**Table 3.**
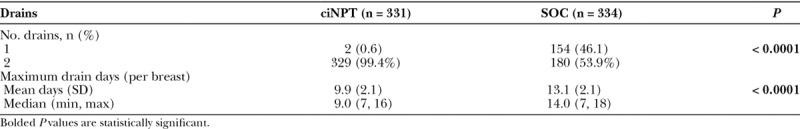
Duration of Drain Use in ciNPT Versus SOC Groups

### Number of Drains and Per Breast Maximum Drain Time

Almost all breasts in the ciNPT group received 2 drains (329/331; 99.4%), whereas 180/334 (53.9%) of the SOC group received 2 drains. The mean number of days from drain placement to removal of the final drain per breast, or “maximum drain days,” was 9.9 days for the ciNPT group and 13.1 days for the SOC group (*P* < 0.0001).

## DISCUSSION

This single site, retrospective analysis compared complication rates and time to drain removal in 356 patients who received ciNPT versus steristrips over 665 closed incisions after breast reconstruction. Significantly lower rates of infection, dehiscence, necrosis, seroma formation, and returns to the OR occurred in the ciNPT group, despite a higher frequency of comorbidities associated with high risk of complications in the ciNPT group. In addition, the mean time to complete removal of all drains was also significantly shorter in the ciNPT group. The per patient cost to the facility for the ciNPT device and dressing used in this study was approximately US $600. Use of ciNPT to actively manage closed breast surgical incisions appeared to play a positive role in minimizing complication rates and drain duration following postmastectomy breast reconstruction in our series, but more study is needed for cost comparison.

Propensity scores have been used in statistics to reduce bias and increase precision in nonrandomized observational studies in many fields, including medical. The technique of propensity score stratification is a way to make an adjustment for covariates before calculating the treatment effect. Exact adjustments made using the propensity score should, on average, remove the bias in the background covariates. In this way, adjustments are made using the estimated propensity scores rather than manipulating all background covariates individually.

To date, this is the largest case series comparing outcomes with ciNPT use versus standard care over closed breast incisions postreconstruction. There are very few studies examining use of ciNPT on closed incisions following breast reconstruction. In a previous retrospective evaluation, our group described the outcomes of 25 patients who received ciNPT over closed incisions following immediate prepectoral breast reconstruction with acellular dermal matrix as part of a 2-stage expander/implant procedure. At the 3-month follow-up, 24/25 (96.0%) breasts healed and remained closed.^27^

Other studies using a ciNPT system that delivers −80 mm Hg or −125 mm Hg have shown positive results in breast incisions. In a retrospective study by Kim et al.^28^, ciNPT at −125 mm Hg was shown to be effective in reducing the incidence of mastectomy flap necrosis in immediate expander-based breast reconstruction. Additionally, results of a prospective study of general surgery patients who received ciNPT at −80 mm Hg (n = 50) or conventional dressings (n = 50) over closed incisions following breast or colorectal surgery showed significantly reduced surgical site events in the ciNPT groups, including in patients over age 65, who comprised at least 40% of the study population.^29^

Preclinical studies involving finite element analysis and bench modeling have demonstrated that the application of ciNPT can reduce the lateral stresses around the incision, alter the direction of the stresses to a distribution more typical of intact tissue, and increase the force needed to disrupt the closed incision by approximately 50%.^30^ This effect of reducing lateral stresses around the incision, in addition to maintaining a closed wound environment, may be beneficial for breast reconstruction patients at high risk for wound dehiscence or infection. To the authors’ knowledge, this is the first study evaluating the effect of ciNPT on the duration of postoperative drains in breast reconstruction patients. Potential benefits of earlier drain removal include shortened time to patient discharge, reduced opportunities for contamination, enhanced patient comfort and convenience, and quicker return to normal activities of daily living. It is possible that micro extractions of exudate may alter the local wound milieu, but more studies are needed to determine the relationship between ciNPT and drain time.

Qualitative data were not formally collected during the study period, but generally, investigators observed that the ciNPT systems seemed well tolerated by patients. Complaints of the therapy were minimal and typical concerns were about the audible motor sounds and leak alarms. To address the leak alarms, patients were instructed to insure negative pressure was intact by making sure the dressing was still collapsed and if necessary applying patching strips to seal the leak (very rare). If the alarm continued, patients were then asked to press the mute button to silence the alert beeps and return with the device to the clinic. Devices were worn around the neck and did not seem to slow patients from returning to their daily activities; patients were able to shower with the dressing in place after disconnecting the therapy unit.

The decision to use xeroform gauze versus steristrips over the closed incision post-ciNPT was based on the preference of the surgeon who removed the ciNPT dressing. During this study time frame, there was no protocol established. Since then, our hospital has established a protocol of leaving the closed incision open to air and applying nothing over it after ciNPT dressing removal.

This study has several limitations. First, the ciNPT group contained older people with greater incidence of diabetes, hypertension, and chemotherapy. All these factors have been shown to be associated with greater risk of postsurgical complications.^13^ Researchers have determined in many cases that patient and preoperative factors are more closely correlated with necrosis and infection than surgical factors.^31^ We have attempted to control for these factors in our analysis using propensity score stratification, but differences between the data sets may still have impacted our outcomes. In addition, duration of drain time may have been impacted by the significantly greater number of breasts in the ciNPT group that received 2 drains, versus 1 drain. Additionally, our results could have been influenced by improvements in surgical techniques and surgical environments over time—between earlier years when steristrips were predominantly used over incisions and most recent years when ciNPT was used. Differential losses to follow-up, variations in patient selection and surgical technique, and absence of data on potential confounding factors are all sources of potential bias in this study. Large, randomized, controlled trials comparing the efficacy and cost-effectiveness of ciNPT versus SOC dressings are needed to add to the body of clinical evidence.

## ACKNOWLEDGMENTS

The authors thank Karen Beach (Acelity) and Rico Martinez (Acelity) for medical writing support. A publication grant was received for this paper from Allergan.
